# Optimal placement and coordination of protective devices in distribution networks considering fuse saving by genetic algorithm

**DOI:** 10.1038/s41598-026-46113-9

**Published:** 2026-04-09

**Authors:** Hossein Ramezani, Mohammad Ebrahim Hajiabadi, Hossein Lotfi, Hossein Parsadust

**Affiliations:** Department of electrical and computer engineering, Hakim Sabzevari university, Sabzevar, Iran

**Keywords:** Radial Distribution Network, Energy Not Supplied (ENS), Optimal Placement, Protective Coordination, Fuse Saving, Genetic Algorithm (GA), Energy science and technology, Engineering, Mathematics and computing

## Abstract

This study proposes a novel framework for the optimal allocation of reclosers and the coordinated operation of protective devices in electrical distribution networks. The main objective is to enhance system reliability and reduce outage related indices by improving the operational performance of reclosers and cutout fuses. Protection settings and Time Current Characteristic (TCC) curves are configured to ensure fast fault detection and effective isolation of faulty sections enabling reclosers to sectionalize the network properly and prevent miscoordination among protection devices. One of the key contributions of the proposed method is the integration of a fuse saving strategy. By assigning priority to reclosers for fault clearing unnecessary fuse operations are avoided which in turn reduces permanent outages. In addition coordination between reclosers and cut out fuses is incorporated into the optimization model in the form of a penalty term within the objective function. This formulation ensures proper selectivity among protective devices and minimizes the network fault response time. To identify the optimal locations and settings of protective equipment a hybrid optimization approach based on graph theory and genetic algorithms has been developed. The proposed method simultaneously considers coordination constraints and fuse-saving requirements. Simulation studies carried out on the IEEE 33-bus test system indicate a considerable improvement in reliability indices. In the second scenario, with four cutout fuses and two reclosers installed, ENS is 50.41% and SAIFI and SAIDI are 46.60% improved compared with the base case. The results demonstrate that reclosers play a fundamental role in mitigating the adverse impacts of faults in distribution systems. Their coordinated operation with cutout fuses together with appropriate adjustment of protection characteristics, significantly enhances network reliability and improves service continuity. Moreover accurate tuning of protective device parameters optimizes fault clearing time and prevents unnecessary operations ultimately leading to improved overall protection system performance.

## Introduction

### Motivation of the study

Since the inception of the electric power industry, ensuring uninterrupted service and providing reliable energy to consumers has always been one of the primary objectives of power systems. Power system professionals have continuously strived to enhance network reliability and stability through ongoing efforts. However, the occurrence of faults remains one of the most significant challenges in achieving this goal particularly in radial overhead distribution networks, which are widely used in both urban and rural areas due to their lower construction and operational costs. Faults in these networks can arise from a variety of factors, including environmental conditions weather events, bird strikes, equipment aging, and other external influences, with a significant portion being transient in nature. If such faults are not managed effectively, they can lead to widespread outages reduced service quality, and customer dissatisfaction. Therefore, the proper deployment and coordination of protective devices, such as reclosers and cutout fuses, is of paramount importance. A key aspect in distribution networks is the fuse-saving strategy. Implementing this concept, which prioritizes fault clearance by reclosers prevents unnecessary fuse operations, thereby extending the lifespan of protective equipment and improving reliability indices. In addition, the optimal placement of protective devices and precise tuning of their time current characteristic (TCC) curves play a vital role in enhancing coordination, minimizing fault clearing time and improving overall network stability. Considering the complexity of distribution networks and the need to simultaneously optimize both device placement and coordination, intelligent optimization algorithms, such as Genetic Algorithms, have proven to be effective tools for this purpose. This approach enables the modeling of coordination constraints, prioritization of fault clearing by reclosers, and reduction of unnecessary fuse operations, ultimately identifying the optimal locations and settings for protective devices within the network. Accordingly the present study aims to propose a comprehensive method for the optimal placement and coordination of protective devices, integrating the fuse-saving concept. The proposed approach contributes directly to improving network reliability and operational performance.

### Review of previous works

#### Optimal placement of protective devices

Ref^1^. -^2^ Replacing traditional Tie Switches (Tie-SWs) with Soft-Open-Points (SOPs) in radial distribution networks can significantly decrease power losses while enhancing voltage and current profiles. The Artificial-Ecosystem-Optimization (AEO) algorithm has demonstrated superior performance compared to Genetic Algorithm (GA) and Particle Swarm Optimization (PSO) in identifying the optimal placement and sizing of SOPs. Ref^3^. A stacked ensemble machine learning model is proposed to determine the optimal number and placement of switches in distribution networks, improving fault management, speeding up service restoration and reducing computational complexity. Ref^4^. With the optimal placement of mechanical switches and the deployment of distributed generation (DG) alongside tie-line switches, the framework enhances the resilience of the distribution network while reducing energy not supplied (ENS). Ref^5^. -^6^ A dual-objective approach for the optimal placement of Telecommunication Load-Breaker Switches (TCLBSs) is proposed, aiming to minimize energy not supplied (ENS) while ensuring the continuity of critical loads, using an iterative graph-based search algorithm. Ref^7^. This study focuses on the optimal placement of automated switches in interconnected microgrids, employing Accelerated Particle Swarm Optimization (APSO) and Genetic Algorithm (GA) to determine the best locations. Ref^8^. This study aims to enhance network reliability by minimizing both consumer power outage costs and switch installation costs, using the Genetic Algorithm (GA) to identify the optimal switch locations. Ref^9^. A method is proposed for optimal recloser placement in distribution networks using the Grey Wolf Optimizer (GWO) to minimize SAIDI and SAIFI. Ref^10^. A method is proposed for the optimal placement of reclosers in distribution networks, employing Monte Carlo simulation and multi-criteria analysis to evaluate reliability indices. Ref^11^. The optimal number and placement of remote-controlled switches and reclosers are determined using Monte Carlo simulation to minimize unserved energy and outage costs under load, fault, and repair uncertainties. Ref^12^. A graph theory based approach is employed to enhance distribution network reliability by optimizing recloser placement to reduce outage related damages. Ref^13^. A graph theory based approach is used to evaluate reliability indices, including SAIFI and SAIDI, by optimally placing reclosers in distribution networks to improve these metrics. Ref^14^. -^15^ This study proposes optimizing the number and placement of reclosers and sectionalizers in distribution networks to improve reliability using the Improved Gravitational Search Algorithm (IGSA). Ref^16^. This study enhances reliability and resilience in distribution systems by deploying remote controlled switches (RCS) and minimizing costs through MILP. Ref^17^. A multi objective optimization approach using NSGA-II is employed to allocate protective devices and switches, considering initial investment and SAIDI. Ref^18^. A hybrid SFLA-PSO algorithm with wavelet mutation is introduced for MOOPF, achieving improved solutions and faster computation. Ref^19^. A hybrid fuzzy CLPSO-DE algorithm is introduced for OAPD with UPFC, lowering costs, maintaining security, and balancing global and local search. Ref^20^. A novel integrated framework leveraging quantum computing and digital twins is introduced to detect and counter FDI attacks in power and transportation systems. Ref^21^. -^22^ presents an adversarial attack framework for DRL based agents in distribution network reconfiguration that disrupts switching operations and overall network performance and proposes a defense method to enhance operational stability and security. Ref^23^. -^24^ a two-stage optimization framework for active distribution networks with hybrid energy storage systems which enhances economic efficiency by optimizing state of charge and employing DRL while managing short term uncertainties of renewable generation.

#### Protective device coordination

Ref^25^. -^26^ A probabilistic analysis of fault currents and overcurrent relay operating times has been carried out to ensure protection coordination in both isolated and grid-connected microgrids. Ref^27^. Optimal settings of dual stage overcurrent relays and appropriate sizing of fault current limiters (FCLs) are applied to ensure fast and reliable protection coordination in both grid-connected and islanded microgrids. Ref^28^. It examines coordination strategies for distance relays in complex transmission lines to ensure both primary and backup protection in modern power systems. Ref^29^. It studies the optimization of recloser and relay coordination in distribution networks to enhance selectivity and shorten fault clearing time. Ref^30^. -^31^ Relay coordination is optimized according to network topology while meeting protection constraints employing a hybrid Metaheuristic Linear Programming algorithm to reduce equipment operation time. Ref^32^. This study examines optimal coordination of protective switches in distribution networks with distributed energy resources, aiming to minimize device operation time through the Genetic Algorithm. Ref^33^. Two methods are introduced for overcurrent device coordination in DG-based distribution networks one using LP with PSO and the other using LP with GA for recloser and fuse coordination. Ref^34^. Focuses on setting protective devices in distribution networks to lower the operation time of protective devices (PD) while keeping protective coordination. Ref^35^. Includes protection coordination constraints as penalties in the objective function, using the Monte Carlo method to optimize coordination among protective devices like relays, reclosers, and fuses. Ref^36^. Proposes an optimal scheme for coordinating reclosers and fuses in radial distribution networks with multiple DGs solved via the Interior Point Method to find optimal settings.

#### Protective coordination and fuse saving

Ref^37^. Investigates protective coordination using the Dual Setting Directional Recloser (DSDR) method, ensuring proper coordination between reclosers and fuses while effectively applying a fuse saving scheme in distribution networks. Ref^38^. Focuses on fuse saving protection in distribution networks by using reclosers to avoid fuse operation during transient faults with recloser and fuse coordination carried out via the Discrete Coordinate Descent (DCD) method. Ref^39^. Investigates protective coordination using two state directional reclosers (DSDR) in distribution networks with distributed generation aiming to coordinate reclosers and fuses while preserving fuse saving mechanisms. Ref^40^. -^41^ Proposes protective coordination in distribution networks with distributed generation (DG), focusing on recloser and fuse coordination and fuse saving through a Multi Agent System (MAS). Ref^42^. Focuses on coordinating reclosers with fuses and Fuse Saving Coordination (FSC) in distribution networks with distributed generation by tuning recloser time coefficients to maintain proper coordination. Ref^43^. Relay operation times are optimized to ensure relay to relay and relay to fuse coordination while applying fuse saving with the Genetic Algorithm used to determine the optimal settings.

In this study, Table 1 compares previous works and the current research in terms of objectives, constraints, and solution methods.

**Table 1:** Comparison of past works and the current work.

Reference	Year	Solution method	Objective Function	Types of Devices	Optimal placement	Protection coordination	Fuse saving
^1^	2025	AEO	Optimal placement of SOPs and Tie-SWs	Tie-SWs, SOPs	**✓**	**-**	-
^2^	2025	Stacked Ensemble Machine Learning	Minimize equipment cost and improve reliability (SAIFI, SAIDI, ENS)	Manual, Automatic Switches	**✓**	-	-
^3^	2025	Distributionally Robust Optimization (DRO)	Optimal placement of remote-controlled switches (RCSs)	RCS	**✓**	-	-
^7^	2024	APSO-GA	Minimizing investment cost, maintenance cost, and customer outage cost	Recloser	**✓**	-	-
^9^	2024	GWO	Minimizing the SAIDI and SAIFI indices	Recloser	**✓**	-	-
^11^	2024	Monte Carlo	Minimizing the ENS and outage costs	Recloser	**✓**	-	-
^13^	2023	Graph theory	Improving reliability indices	Recloser	**✓**	-	-
^14^	2023	IGSA	Reducing ENS and improving reliability indices	Recloser, sectionalizer	**✓**	-	-
^16^	2024	MILP	Minimizing investment costs, maintenance costs, and customer outage costs	RCS	**✓**	-	-
^25^	2025	Monte Carlo	Protective coordination relays	Relay	-	**✓**	-
^27^	2025		Minimization of relay operating time	Relay	-	**✓**	-
^32^	2022	GA	Protective coordination of reclosers and fuses	Recloser, Fuse	-	**✓**	-
^33^	2022	PSO، GA	Minimizing the operation time of equipment and ensuring coordination between them	Recloser, Fuse	-	**✓**	-
^34^	2020	GA	Minimizing the operation time of equipment and coordinating them effectively	Recloser, Relay	-	**✓**	-
^35^	2020	Monte Carlo	Protective coordination of equipment	Recloser Relay, Fuse	-	**✓**	-
^36^	2018	IPM	Protective coordination of reclosers and fuses	Recloser, Fuse	-	**✓**	-
^37^	2021	GA	Protective coordination of reclosers and fuses	Recloser, Fuse	-	**✓**	✓
^38^	2024	DCD	Protective coordination of reclosers and fuses with fuse-saving	Recloser, Fuse	-	**✓**	✓
^39^	2022	GA	Protective coordination of reclosers and fuses with fuse-saving	Recloser, Fuse	-	**✓**	✓
^40^	2023	MAS	Protective coordination of reclosers and fuses with fuse-saving	Recloser, Fuse	-	**✓**	✓
^42^	2024	GA	Protective coordination of reclosers and fuses with fuse-saving	Recloser, Fuse	-	**✓**	✓
^43^	2024	GA	Protective coordination of equipment with fuse-saving	Relay, Fuse	-	**✓**	✓
Proposed		GA	Minimizing reliability indices (ENS, SAIFI and SAIDI)	Recloser, Fuse	**✓**	**✓**	✓

### Contributions

Considering that in references [1–6] the optimal placement of protective devices has been investigated with the aim of improving reliability indices in references [20–26] protective coordination among devices has been studied without accounting for optimal placement and in references [30–35] protective coordination based on the Fuse Saving concept has been examined a clear research gap is observed in the existing literature. In other words to date no comprehensive framework has been proposed that simultaneously considers the optimal placement and setting of protective devices protective coordination and the Fuse Saving concept with the goal of improving network reliability indices. The key contributions of this study have a significant impact on enhancing the performance and efficiency of protective systems which are detailed below:

- One of the key achievements of this study is the optimal placement of reclosers in radial distribution networks. Considering the wide geographical coverage and structural complexity of distribution systems, strategically selecting recloser installation points plays a crucial role in improving reliability indices. The proposed hybrid approach, based on graph theory and genetic algorithms, is designed to simultaneously optimize both the locations and the setting parameters of protective devices. This enables rapid detection and clearance of transient faults and prevents widespread outages. Overall, this innovation enhances network reliability and increases customer satisfaction.
- A novel and precise modeling of protective coordination between reclosers and cut-out fuses is introduced. This coordination is incorporated as a penalty constraint within the objective function, providing greater flexibility in protective device settings. It enables achieving optimal settings aimed at minimizing fault clearance time, avoiding protective device operation interference, and improving supply continuity. The penalty-based modeling ensures effective coordination between reclosers and fuses throughout the network.
- The fuse saving concept prevents unnecessary fuse operations during transient faults by allowing reclosers to act quickly first with fuses operating only if the fault persists. This strategy not only significantly extends the lifespan of protective equipment but also substantially reduces maintenance and replacement costs for fuses. Moreover it decreases unnecessary outages and improves power quality for consumers. In this study the fuse saving concept is comprehensively integrated into the optimization framework and its impact on enhancing network protection performance is thoroughly evaluated.

The paper is organized as follows, Section two introduces the objective functions and constraints, section three presents the solution method, and sections four and five provide the simulation results and conclusions.

## Problem formulation

This section is divided into multiple parts, each covering a specific aspect of the study. The first subsection presents the power flow formulation using a graph based approach offering a structured view of the system. The second subsection addresses protection formulation highlighting the key considerations and applied methods. The third and fourth subsections introduce the objective function and protective constraints respectively explaining their roles in ensuring system reliability and operational security.

### Power flow formulation based on graph

A network with *N* nodes and *L* branches can be define by graph *G* with the incidence matrix *T* and the adjacency matrix *M*:

1 $$T={[{t_{n,l}}]_{N \times L}}\begin{array}{*{20}{c}} {}&{{\text{node and line incidence matrix}}}&{} \end{array}$$

$${t_{n,l}}=\left\{ {\begin{array}{*{20}{c}} {1\begin{array}{*{20}{c}} {}&{\begin{array}{*{20}{c}} {{\mathrm{if}}\begin{array}{*{20}{c}} {{\mathrm{Line}}\begin{array}{*{20}{c}} {l\begin{array}{*{20}{c}} {{\mathrm{is}}\begin{array}{*{20}{c}} {{\mathrm{coonected}}\begin{array}{*{20}{c}} {{\mathrm{to}}\begin{array}{*{20}{c}} {{\mathrm{node}}\begin{array}{*{20}{c}} {\mathrm{n}}&{} \end{array}}&{} \end{array}}&{} \end{array}}&{} \end{array}}&{} \end{array}}&{} \end{array}}&{} \end{array}}&{} \end{array}} \end{array}} \\ {0\begin{array}{*{20}{c}} {\begin{array}{*{20}{c}} {\begin{array}{*{20}{c}} {\begin{array}{*{20}{c}} {\begin{array}{*{20}{c}} {}&{\begin{array}{*{20}{c}} {}&{} \end{array}} \end{array}}&{}&{{\mathrm{otherwise}}} \end{array}}&{}&{} \end{array}\begin{array}{*{20}{c}} {\begin{array}{*{20}{c}} {\begin{array}{*{20}{c}} {\begin{array}{*{20}{c}} {}&{} \end{array}}&{} \end{array}}&{} \end{array}}&{} \end{array}}&{}&{}&{} \end{array}}&{} \end{array}} \end{array}} \right.$$

2 $$M={[{m_{i,j}}]_{N \times N}}=sign(T \times T')$$

$${m_{i,j}}=\left\{ {\begin{array}{*{20}{c}} {1\begin{array}{*{20}{c}} {}&{\begin{array}{*{20}{c}} {{\mathrm{if}}\begin{array}{*{20}{c}} {{\mathrm{node}}\begin{array}{*{20}{c}} {i\begin{array}{*{20}{c}} {{\text{ is}}\begin{array}{*{20}{c}} {{\mathrm{coonected}}\begin{array}{*{20}{c}} {{\mathrm{to}}\begin{array}{*{20}{c}} {{\mathrm{node}}\begin{array}{*{20}{c}} {{\text{j directly}}}&{} \end{array}}&{} \end{array}}&{} \end{array}}&{} \end{array}}&{} \end{array}}&{} \end{array}}&{} \end{array}}&{} \end{array}} \end{array}} \\ {0\begin{array}{*{20}{c}} {\begin{array}{*{20}{c}} {\begin{array}{*{20}{c}} {\begin{array}{*{20}{c}} {\begin{array}{*{20}{c}} {}&{\begin{array}{*{20}{c}} {}&{} \end{array}} \end{array}}&{}&{{\mathrm{otherwise}}} \end{array}}&{\begin{array}{*{20}{c}} {\begin{array}{*{20}{c}} {}&{} \end{array}}&{} \end{array}}&{} \end{array}\begin{array}{*{20}{c}} {\begin{array}{*{20}{c}} {\begin{array}{*{20}{c}} {\begin{array}{*{20}{c}} {}&{} \end{array}}&{} \end{array}}&{} \end{array}}&{} \end{array}}&{}&{}&{} \end{array}}&{} \end{array}} \end{array}} \right.$$

3 $$\begin{gathered} {M_n}={[m_{{i,j}}^{n}]_{N \times N}}=sign({M_{n - 1}} \times {M_1})\begin{array}{*{20}{c}} {}&{} \end{array}n=2,...,N \hfill \\ {M_1}=M \hfill \\ \end{gathered}$$

For each radial distribution network with specified tree graph *G* and supplying point *S* the following Matrices can be define: Downstream Node.

Down Warding Node Matrix (*DWNM*): *DWNM* is a *L×N* matrix, in which row *l* determines the set of nodes which will be isolated from *S* if line *l* is removed from *G*. These nodes are considered as the down warding nodes of line *l*. Equation (4) defines the elements of *DWNM*.

4 $$DWNM(l,n)=\left\{ {\begin{array}{*{20}{c}} {1\begin{array}{*{20}{c}} {}&{\begin{array}{*{20}{c}} {{\mathrm{if}}\begin{array}{*{20}{c}} {{\mathrm{node}}\begin{array}{*{20}{c}} {{\mathrm{n}}\begin{array}{*{20}{c}} {{\mathrm{is}}\begin{array}{*{20}{c}} {{\mathrm{isolated}}\begin{array}{*{20}{c}} {\begin{array}{*{20}{c}} {\begin{array}{*{20}{c}} {{\mathrm{from}}\begin{array}{*{20}{c}} {{\mathrm{S}}\begin{array}{*{20}{c}} {{\mathrm{in}}\begin{array}{*{20}{c}} {{{\mathrm{G}}_{\mathrm{l}}}}&{} \end{array}}&{} \end{array}}&{} \end{array}}&{} \end{array}}&{} \end{array}}&{} \end{array}}&{} \end{array}}&{} \end{array}}&{} \end{array}}&{} \end{array}}&{} \end{array}} \end{array}} \\ {0\begin{array}{*{20}{c}} {\begin{array}{*{20}{c}} {\begin{array}{*{20}{c}} {\begin{array}{*{20}{c}} {\begin{array}{*{20}{c}} {}&{\begin{array}{*{20}{c}} {}&{} \end{array}} \end{array}}&{}&{{\mathrm{otherwise}}} \end{array}}&{\begin{array}{*{20}{c}} {\begin{array}{*{20}{c}} {\begin{array}{*{20}{c}} {}&{} \end{array}}&{} \end{array}}&{} \end{array}}&{} \end{array}\begin{array}{*{20}{c}} {\begin{array}{*{20}{c}} {\begin{array}{*{20}{c}} {\begin{array}{*{20}{c}} {}&{} \end{array}}&{} \end{array}}&{} \end{array}}&{} \end{array}}&{}&{}&{} \end{array}}&{} \end{array}} \end{array}} \right.$$

According to the Corollary, each row of *DWNM* is calculated using the adjacency matrix$M_{l}^{{N - 1}}$

5 $$DWNM(l,:)=1 - M_{l}^{{N - 1}}(S,:)$$

6 $$DWNM(:,S)=0$$

The *BIBC* (bus injection to branch current) matrix shows the relationship between the injected currents in the network buses and the flowing currents in its branches.

7 $$BIB{C_{l,n}}=DWNM$$

The following equation shows the impedance value of each line. *R* is lines resistance and *X* is lines reactance.

8 $${Z_{1,l}}={R_{1,l}}+j{X_{1,l}}$$

In the first step, the voltage of all nodes is considered to be 1 per unit.

9 $${V_o}={[{I_{n,1}}]_{N \times 1}}\begin{array}{*{20}{c}} {}&{{I_{n,1}}=1} \end{array}\begin{array}{*{20}{c}} {;{\mathrm{Always}}\begin{array}{*{20}{c}} {{\mathrm{is}}\begin{array}{*{20}{c}} 1&{} \end{array}}&{} \end{array}}&{} \end{array}$$

10 $$BCB{V_{n,l}}={(BIB{C_{l,n}})^T} \odot ({V_0} \times {Z_{1,l}})$$

The *BCBV* (branch current to bus voltage) matrix is representing the relationship between the branch currents and the bus voltages. Using the *BCBV* matrix, the respective variation of the bus voltages which is generated by the variation of the branch currents is established directly.

The following equations are used to calculate the loaf current of each bus, where *S* is the apparent power, *P* is the active power, and *Q* is the reactive power of each bus.

11 $${S_{n,1}}={P_{n,1}}+j{Q_{n,1}}$$

12 $${[{I_n}]_{n,1}}=\frac{{S_{{n,1}}^{*}}}{{V_{0}^{*}}}$$

Then, using Eqs. (13), (14), the current passing through each line and the voltage of the nodes are calculated.

13 $${[{I_b}]_{l,1}}=BIB{C_{l,n}} \times {I_n}$$

14 $$~{[Vn]_{n,1}}={V_{n - 1}} - BCB{V_{n,l}} \times {I_b}$$

In the power flow program, Eqs. (15) and (16) must be satisfied for bus voltage convergence; otherwise, the power flow calculations will be repeated iteratively in a forward and backward manner.

15 $$Error=\left| {{V_n} - {V_{n - 1}}} \right|\langle \varepsilon$$

16 $$0.9p.u\langle \left| {{V_n}} \right|\langle 1p.u$$

### Protection formulation

This section presents the protection formulation addressing key concepts such as short circuit current calculation, pickup current, cut out fuses, relay operating times and recloser-fuse coordination including both fast and slow recloser operations, These topics are discussed in detail in the following subsections.

#### Short-circuit current calculations

The short-circuit current at each bus is calculated using Eq. (17). For this analysis, the fault impedance *Z*_*f*_ is assumed to be zero.

17 $$\begin{gathered} {[I_{k}^{f}]_{N \times 1}}=\frac{{{{[{V_n}]}_{N \times 1}}}}{{(diag{{[{Z_{kk}}]}_{N \times N}})+{Z_f}}}\mathop {}\limits_{{}}^{{}} \mathop {}\limits_{{}}^{{}} \mathop {}\limits_{{}}^{{}} \mathop {}\limits_{{}}^{{}} ,diag{[{Z_{kk}}]_{N \times N}}=\left[ \begin{gathered} {Z_{11}} \hfill \\ _{{}} \vdots \hfill \\ _{{}} \vdots \hfill \\ {Z_{kk}} \hfill \\ \end{gathered} \right] \hfill \\ \hfill \\ \end{gathered}$$

In this context, ${[I_{k}^{f}]_{N \times 1}}$ represents the short-circuit current at each bus *k*. The pre-fault voltages are obtained using a forward-backward load flow analysis. For a fault occurring at bus k, the calculation requires the ${Z_{kk}}$element of the impedance matrix, which represents the Thevenin equivalent impedance as seen from the faulted bus. Using Eq. (18), the voltages at the buses during the fault are calculated.

18 $${[V_{n}^{f}]_{N \times N}}={[{V_n}]_{N,1}} - {Z_n}_{k} \times {[I_{k}^{f}]_{N,1}}$$

In this equation,${[V_{n}^{f}]_{N \times N}}$is the voltage of bus *n* during the fault, and${[{V_n}]_{N,1}}$is the voltage of bus n before the fault. Based on Eq. (19), the currents of all lines are calculated.

19 $${[{I_s}_{c}]_{n,n+1}}=\frac{{{{[V_{n}^{f}]}_n} - {{[V_{n}^{f}]}_{n+1}}}}{{{Z_{1,l}}}}$$

In this equation,${Z_{1,l}}$represents the impedance of the line between two buses.${[V_{n}^{f}]_n}$and${[V_{n}^{f}]_{n+1}}$represents the bus voltages during the fault.

#### Pickup current

The pickup current for reclosers and fuse selection should not be lower than the load current. Typically, it is considered as 1.3 times the load current, as shown in Eq. (20) [44].

20 $${I_{pickup}}=1.3 \times {[{I_b}]_{l,1}}$$

In this equation, ${[{I_b}]_{l,1}}$represents the load current of the lines and ${I_{pickup}}$ denotes the pickup current.

#### Cut-out fuse

Fuses melt at a specific fault current and within a defined time. The current-time characteristic of a fuse is represented by two curves: the Minimum Melting Time (*MMT*) curve and the Maximum Clearing Time (*MCT*) curve. Based on their operation, fuses are categorized into two types: slow-blow (*T*) and fast-blow (*K*).

#### Relay operating time

The relay operating time is determined based on IEEE and IEC standards and is presented in Eqs. (21) and (22) [45]- [46].

21 $$O{T^{ieee}}({I_{sc}})=TDS \times (\frac{A}{{{{({{I_{{sc}}^{{}}} \mathord{\left/ {\vphantom {{I_{{sc}}^{{}}} {{I_{pickup}}}}} \right. \kern-0pt} {{I_{pickup}}}})}^P} - 1}}+B)$$

22 $$O{T^{iec}}({I_{sc}})=TDS \times (\frac{A}{{{{({{I_{{sc}}^{{}}} \mathord{\left/ {\vphantom {{I_{{sc}}^{{}}} {{I_{pickup}}}}} \right. \kern-0pt} {{I_{pickup}}}})}^P} - 1}})$$

In these equations, $\:{I}_{sc}$ represents the short-circuit current detected by the relay, $\:{I}_{pickup}\:$is the relay pickup current, *TDS* denotes the relay time dial setting, *A*, *B*, and *P* are fixed parameters that define the type of the relay curve.

#### Recloser-fuse coordination

##### Fast operation of the recloser

The fast operation time of recloser *R*_*j*_ must be shorter than the minimum melting time of fuse *Cut*_*i*_. Naturally, the fast operation time of the recloser should be coordinated with all downstream cut-out fuses. The fast operation time of the recloser, based on IEEE and IEC standards, is given in Eqs. (23) and (24).

23 $$OT_{{{\operatorname{R} _j}}}^{{ieee,Fast}}({I_{sc}})=TDS_{{{\operatorname{R} _j}}}^{{ieee,Fast}} \times (\frac{A}{{{{({{I_{{sc,max}}^{{^{{cu{t_i}}}}}} \mathord{\left/ {\vphantom {{I_{{sc,max}}^{{^{{cu{t_i}}}}}} {{I_{pickup}}}}} \right. \kern-0pt} {{I_{pickup}}}})}^P} - 1}}+B)$$

24 $$OT_{{{\operatorname{R} _j}}}^{{iec,Fast}}({I_{sc}})=TDS_{{{\operatorname{R} _j}}}^{{iec,Fast}} \times (\frac{A}{{{{({{I_{{sc,max}}^{{^{{cu{t_i}}}}}} \mathord{\left/ {\vphantom {{I_{{sc,max}}^{{^{{cu{t_i}}}}}} {{I_{pickup}}}}} \right. \kern-0pt} {{I_{pickup}}}})}^P} - 1}})$$

The fast operation of recloser *j* before the minimum melting time of fuse *i* enables fuse saving, as shown in Eq. (25).

25 $$OT_{{_{{{R_j}}}}}^{{Fast}}(I_{{sc,max}}^{{^{{cu{t_i}}}}})=(MM{T_i} - CTI_{{{R_j}}}^{{Fast}}) \times F{S^i}$$

$\:{I}_{{SC,max}_{i}}^{{Cut}_{i}}\:$represents the maximum fault current passing through fuse $\:{Cut}_{i}$, which occurs during a short-circuit fault with $\:{Z}_{SC}=0\:$in front of $\:{Cut}_{i}$. $\:{MMT}_{i}\:$denotes the minimum melting time of fuse *i*, $CTI_{{{R_j}}}^{{Fast}}$ is the coordination time interval for the recloser in fast mode, and $\:{FS}^{i}\:$represents the fuse-saving condition for fuse *i*.

If the minimum melting time of the fuse is greater than zero, it must be coordinated with the fast operation of the recloser. However, if the fuse melting time is zero, fuse-saving does not apply, as shown in Eq. (26).

26 $$F{S^i}=\left\{ {\begin{array}{*{20}{c}} {1\begin{array}{*{20}{c}} {}&{\begin{array}{*{20}{c}} {{\mathrm{If}}}&{\begin{array}{*{20}{c}} {{\text{Coordination of Recloser With Fuse}}}&{} \end{array}} \end{array}} \end{array}} \\ {0\begin{array}{*{20}{c}} {\begin{array}{*{20}{c}} {\begin{array}{*{20}{c}} {\begin{array}{*{20}{c}} {\begin{array}{*{20}{c}} {}&{\begin{array}{*{20}{c}} {}&{} \end{array}} \end{array}}&{}&{{\mathrm{otherwise}}} \end{array}}&{}&{} \end{array}}&{}&{}&{} \end{array}}&{} \end{array}} \end{array}} \right.$$

The time dial setting $TDS_{{{R_j}}}^{{Fast}}$ for recloser *Rj* in fast mode is obtained from the results of Eqs. (23) and (25) and is presented in Eq. (27).

27 $$TDS_{{{R_j},Cu{t_i}}}^{{Fast}}={{(MMT_{{Isc,max}}^{{Cu{t_i}}} - CTI_{{{R_j}}}^{{Fast}})} \mathord{\left/ {\vphantom {{(MMT_{{Isc,max}}^{{Cu{t_i}}} - CTI_{{{R_j}}}^{{Fast}})} {(\frac{A}{{{{({{I_{{sc,max}}^{{^{{cu{t_i}}}}}} \mathord{\left/ {\vphantom {{I_{{sc,max}}^{{^{{cu{t_i}}}}}} {I_{{pickup}}^{{{R_j}}}}}} \right. \kern-0pt} {I_{{pickup}}^{{{R_j}}}}})}^P} - 1}}+B)}}} \right. \kern-0pt} {(\frac{A}{{{{({{I_{{sc,max}}^{{^{{cu{t_i}}}}}} \mathord{\left/ {\vphantom {{I_{{sc,max}}^{{^{{cu{t_i}}}}}} {I_{{pickup}}^{{{R_j}}}}}} \right. \kern-0pt} {I_{{pickup}}^{{{R_j}}}}})}^P} - 1}}+B)}}$$

For all cut out fuses with$F{S^i}=1$, the fast operation of the recloser must occur before the minimum melting time of the fuse, as shown in Eq. (28).

28 $$\left\{ \begin{gathered} \forall \begin{array}{*{20}{c}} {}&{Cu{t^i}} \end{array}_{{}}^{{}}\therefore _{{}}^{{}}F{S^i}=1 \hfill \\ \forall \begin{array}{*{20}{c}} {}&{OT_{{{R_j}}}^{{Fast}}} \end{array}(I_{{sc,max}}^{{Cu{t_i}}})_{{}}^{{}}\langle _{{}}^{{}}MM{T_i} - CTI_{{{R_j}}}^{{Fast}} \hfill \\ \end{gathered} \right.$$

In Eq. (29), $TDS_{{{R_j}}}^{{Fast}}$ represents the time dial setting for the fast operation of recloser *R*_*j*_ before the minimum melting time of fuse *Cut*_*i*_. The minimum value of this time for fuses with fuse-saving is determined based on the minimum melting time curve of the fuse.

29 $$TDS_{{{R_j}}}^{{Fast}}=\mathop {min}\limits_{{i,F_{i}^{j} \ne 0}} (TDS_{{{R_j},Cu{t_i}}}^{{Fast}})$$

##### Slow operation of the recloser

The slow operation time of recloser *R*_*j*_ occurs after the maximum clearing time of fuse *Cut*_*i*_. Naturally, the slow operation time of the recloser must be coordinated with all downstream cut-out fuses. The slow operation time of the recloser, according to IEEE and IEC standards, is given in Eqs. (30) and (31).

30 $$OT_{{{\operatorname{R} _j}}}^{{ieee,Slow}}({I_{sc}})=TDS_{{{\operatorname{R} _j}}}^{{ieee,Slow}} \times (\frac{A}{{{{({{I_{{sc,max}}^{{^{{cu{t_i}}}}}} \mathord{\left/ {\vphantom {{I_{{sc,max}}^{{^{{cu{t_i}}}}}} {{I_{pickup}}}}} \right. \kern-0pt} {{I_{pickup}}}})}^P} - 1}}+B)$$

31 $$OT_{{{\operatorname{R} _j}}}^{{iec,Slow}}({I_{sc}})=TDS_{{{\operatorname{R} _j}}}^{{iec,Slow}} \times (\frac{A}{{{{({{I_{{sc,max}}^{{^{{cu{t_i}}}}}} \mathord{\left/ {\vphantom {{I_{{sc,max}}^{{^{{cu{t_i}}}}}} {{I_{pickup}}}}} \right. \kern-0pt} {{I_{pickup}}}})}^P} - 1}})$$

The slow operation of recloser *Rj* after the maximum clearing time of fuse *Cut*_*i*_ results in isolating the fault location, as shown in Eq. (32).

32 $$OT_{{_{{{R_j}}}}}^{{Slow}}(I_{{sc,max}}^{{^{{cu{t_i}}}}})=(MC{T_i} - CTI_{R}^{{Slow}}) \times F{S^i}$$

The time dial setting$TDS_{{{R_j}}}^{{Slow}}$for recloser *Rj* in slow mode is obtained from the results of Eqs. (30) and (32) and is presented in Eq. (33).

33 $$TDS_{{{R_j},Cu{t_i}}}^{{Slow}}={{(MCT_{{Isc,max}}^{{Cu{t_i}}} - CTI_{{{R_j}}}^{{Slow}})} \mathord{\left/ {\vphantom {{(MCT_{{Isc,max}}^{{Cu{t_i}}} - CTI_{{{R_j}}}^{{Slow}})} {(\frac{A}{{{{({{I_{{sc,max}}^{{^{{cu{t_i}}}}}} \mathord{\left/ {\vphantom {{I_{{sc,max}}^{{^{{cu{t_i}}}}}} {I_{{pickup}}^{{{R_j}}}}}} \right. \kern-0pt} {I_{{pickup}}^{{{R_j}}}}})}^P} - 1}}+B)}}} \right. \kern-0pt} {(\frac{A}{{{{({{I_{{sc,max}}^{{^{{cu{t_i}}}}}} \mathord{\left/ {\vphantom {{I_{{sc,max}}^{{^{{cu{t_i}}}}}} {I_{{pickup}}^{{{R_j}}}}}} \right. \kern-0pt} {I_{{pickup}}^{{{R_j}}}}})}^P} - 1}}+B)}}$$

In Eq. (34), $TDS_{{{R_j}}}^{{Slow}}$represents the time dial setting for the slow operation of recloser *R*_*j*_ after the maximum clearing time of fuse *Cut*_*i*_. The maximum value of this time for fuses with fuse saving is determined based on the maximum melting time curve of the fuse.

34 $$TDS_{{{R_j}}}^{{Slow}}=\mathop {max}\limits_{{i,F_{i}^{j} \ne 0}} (TDS_{{{R_j},Cu{t_i}}}^{{Slow}})$$

### Objective function

The objective function for the optimal placement of reclosers in the network is defined based on minimizing reliability indices. The constraint related to determining the operational range of the equipment to coordinate with the upstream device is incorporated as a penalty in the objective function, as shown in Eq. (35) [47].

35 $$\mathop {OF=\mathop {min}\limits_{{{R_L}}} }\limits_{{}} \begin{array}{*{20}{c}} {}&{\left( {{W_1} \times \frac{{ENS}}{{EN{S_{base}}}}+{W_2} \times \frac{{SAIFI}}{{SAIF{I_{base}}}}+{W_3} \times \frac{{SAIDI}}{{SAID{I_{base}}}}} \right)} \end{array}+Penalty$$

Equation (35) defines the objective function based on reliability indices, including the System Average Interruption Frequency Index (SAIFI) the System Average Interruption Duration Index (SAIDI) and the Energy Not Supplied (ENS). In this formulation, the weighting factors W1, W2, and W3 are considered equal (W1 = W2 = W3 = 0.33) to ensure that each index contributes equally to the objective function.

In this study, the control variable of the optimization problem is clearly defined within the objective function and the decision making model. The vector $\:{R}_{L\:}$is considered a binary decision variable that specifies whether a recloser is installed on the distribution network lines. Each component $\:{R}_{L\:}$can take only one of two values, zero or one: a value of one indicates that a recloser is installed on line $\:l$, while a zero represents the absence of a recloser on that line.

This variable plays a fundamental role in the model, as changes in $\:{R}_{L}\:$lead to variations in the outage duration of load nodes $\:{U}_{n}\left({R}_{L}\right)$ which consequently affect the total energy not supplied ENS. Moreover, optimizing the values of $\:{R}_{L}$ enables the optimization algorithm to determine both the optimal placement and operational strategy of the reclosers in a way that minimizes the overall ENS and enhances the reliability of the distribution system. Therefore, the vector $\:{R}_{L}$serves as the main control variable of the problem and directly influences the objective function.

36 $${R_L}=\left\{ {\begin{array}{*{20}{c}} {1\begin{array}{*{20}{c}} {\begin{array}{*{20}{c}} {}&{}&{} \end{array}{\text{If Installation of a recloser on line l}}}&{}&{}&{} \end{array}} \\ {0\begin{array}{*{20}{c}} {\begin{array}{*{20}{c}} {\begin{array}{*{20}{c}} {\begin{array}{*{20}{c}} {\begin{array}{*{20}{c}} {\begin{array}{*{20}{c}} {}&{} \end{array}}&{{\mathrm{otherwise}}}&{}&{} \end{array}}&{}&{} \end{array}}&{} \end{array}}&{} \end{array}}&{}&{\begin{array}{*{20}{c}} {}&{} \end{array}}&{} \end{array}} \end{array}} \right.$$

The amount of Energy Not Supplied in the network, which is reduced through the optimal placement of reclosers is measured in kilowatt-hours per year. It is calculated using Eq. (37).

37 $$ENS=\sum\limits_{n} {{U_n}({R_L}) \times {L_n}}$$

${U_n}({R_L})$represents the outage duration at load point $\:n$over a specified period (one year) and is a function of the decision vector${R_L}$.In other words, the installation or absence of reclosers on the network lines directly affects the outage duration of each load point.${L_n}$denotes the load demand at point $\:n$, which is multiplied by the outage duration to calculate the energy not supplied in kWh at each load point. By optimally selecting the values of${R_L}$, the outage durations${U_n}({R_L})$are reduced, resulting in a decrease in the total ENS of the network. This relationship clearly demonstrates that decisions regarding recloser placement (the control variable${R_L}$) directly impact the performance and reliability of the distribution system, as shown in Eq. (38).

38 $${U_n}({R_L})=\sum\limits_{{}} {{U_{l,n}}{X_{l,n}}({R_L})}$$

${X_{l,n}}$is one of the key parameters in the reliability analysis of distribution networks and represents the functional relationship between line $\:l\:$and point $\:n$. Specifically, ${X_{l,n}}$determines whether the failure of line $\:l\:$will cause an outage at load point $\:n$. From a mathematical modeling perspective, ${X_{l,n}}$is defined as a binary variable [48]:

39 $${X_{l,n}}=\left\{ {\begin{array}{*{20}{c}} {1\begin{array}{*{20}{c}} {}&{{\text{if the failure of line l results in a load outage at point n}}_{{}}^{{}}} \end{array}} \\ {0\begin{array}{*{20}{c}} {\begin{array}{*{20}{c}} {\begin{array}{*{20}{c}} {\begin{array}{*{20}{c}} {\begin{array}{*{20}{c}} {\begin{array}{*{20}{c}} {\begin{array}{*{20}{c}} {\begin{array}{*{20}{c}} {}&{{\mathrm{otherwise}}} \end{array}}&{} \end{array}}&{} \end{array}}&{}&{}&{} \end{array}}&{}&{} \end{array}}&{} \end{array}}&{} \end{array}}&{}&{}&{} \end{array}} \end{array}} \right.$$

In this model, the distribution network is represented as a connected graph consisting of nodes (load points and generation sources) and edges (lines). From the perspective of graph theory and topological network analysis, ${X_{l,n}}$indicates whether the power supply path from the sources to load point $\:n\:$passes through line $\:l$. If line $\:l\:$fails and no alternative path exists to supply the load, ${X_{l,n}}$is assigned a value of 1, representing the critical impact of that line on the load. Otherwise, its value is 0.

*U*_*l, n*_ is the average outage duration of load point n due to the failure of line l. Additionally${t_l}_{{,n}}$Repair time of line $\:l$ restore service to load point n and${\lambda _l}$represents the failure rate of the line.

40 $${U_{l,n}}={\lambda _l} \times {t_l}_{{,n}}$$

41 $${t_{l,n}}=\left\{ {\begin{array}{*{20}{c}} {\begin{array}{*{20}{c}} {{r_t}_{{,l}}}&{\begin{array}{*{20}{c}} {{\text{line repair tim}}{{\mathrm{e}}_{}}l}&{\begin{array}{*{20}{c}} {\begin{array}{*{20}{c}} {}&{} \end{array}}&{}&{}&{} \end{array}} \end{array}} \end{array}} \\ {\begin{array}{*{20}{c}} {\begin{array}{*{20}{c}} {St}&{\begin{array}{*{20}{c}} {{\text{Switch time}}}&{}&{} \end{array}}&{}&{} \end{array}}&{}&{}&{} \end{array}} \end{array}} \right.$$

The outage duration of load points caused by line failures consists of two main components: the line repair time ($\:{r}_{t,l}$) and the protective device switching time ($\:St$). The line repair time ($\:{r}_{t,l}$) represents the time required to repair the fault on line $\:l\:$and restore service to load point $\:n$. The switching time ($\:St$) refers to the duration that protective devices, such as switches and reclosers, take to operate for isolating the faulted section and restoring power to unaffected load point s as quickly as possible.

Therefore the reliability indices, System Average Interruption Frequency Index (SAIFI) and System Average Interruption Duration Index (SAIDI) are defined for each load point as follows:

42 $$SAIFI=\frac{{\sum\nolimits_{n} {{N_n} \times {\lambda _n}} }}{{\sum\nolimits_{n} {{N_n}} }}$$

43 $$SAIDI=\frac{{\sum\nolimits_{n} {{N_n} \times {U_n}} }}{{\sum\nolimits_{n} {{N_n}} }}$$

where $\:{\lambda\:}_{n}$ represents the average annual permanent fault rate at load point $\:n$. $\:{U}_{n}\:$and $\:{N}_{n\:}$respectively denote the average duration of permanent outages at point $\:n$and the number of customers connected to point $\:n$.

### Coordination constraint

The protective coordination constraint for the recloser and fuse has been implemented as a penalty in the objective function of this study. For fuses with fuse-saving capability, the recloser in fast mode must operate before the minimum melting time of the fuse in the event of a downstream short circuit to protect the fuse, as shown in Eq. (44).

44 $$Penalty=OT_{{{R_j}}}^{{Fast}}{(I_{{sc,max}}^{{Cu{t_i}}})_{}}{\langle _{}}MM{T_i} - CTI_{{{R_j}}}^{{Fast}}$$

In this equation, the protection constraint ensures the coordination between the recloser and fuse in the network. The operating time of the recloser at the maximum short-circuit current upstream of the fuse $OT_{{{R_j}}}^{{Fast}}(I_{{sc,max}}^{{Cu{t_i}}})$is compared with the sum of the minimum melting time of the fuse $MM{T_i}$ and the coordination time between the recloser and fuse $CTI_{{{R_j}}}^{{Fast}}$. If the recloser’s operating time exceeds this value, meaning it operates later than the fuse, the fuse will blow. In such a case, a penalty is applied in the objective function to make this solution undesirable in the optimization process. The purpose of this constraint is to ensure that the recloser operates faster than the minimum melting time of the fuse under downstream short-circuit conditions, enabling fuse saving. This approach enhances network reliability, improves recloser-fuse coordination, and facilitates fuse saving.

## Solution method

To effectively solve the problem of optimal protective device placement with fuse-saving coordination, we employed the Genetic Algorithm (GA) a well-established evolutionary technique inspired by the principles of natural selection and genetics. GA is particularly well-suited for solving complex optimization problems in power systems where the search space is large, nonlinear, and discrete, and where traditional methods often fail due to the presence of non-differentiable or discontinuous objective functions. Chromosome Representation In this study, each solution (or chromosome) is represented as a vector of integers where each integer corresponds to a specific location in the distribution network where a recloser can be placed. For example a chromosome like [6,25] indicates that reclosers are to be installed at Lines 6 and 25. This encoding approach enables the algorithm to flexibly explore different combinations of device placements across the network.

### Initial population

An initial population of chromosomes is randomly generated, ensuring a diverse set of candidate solutions. However to increase the algorithms efficiency we incorporate domain specific knowledge into this step for example avoiding locations where reclosers cannot be physically installed or where they would conflict with existing fuse positions.

### Objective function

Based on Eq. (35), the main objective of this study is to minimize the reliability indices, while also maintaining proper coordination between reclosers and fuses.

Here the reliability indices are calculated based on the systems load data and fault probabilities. penalty​ represents a penalty term for any violations in protection coordination logic e.g., scenarios where a fuse blows unnecessarily instead of the upstream recloser operating. This formulation ensures that the algorithm favors solutions that not only reduce outages but also extend equipment life by preserving fuses through the fuse saving approach.

### Genetic operators

Once the initial population is evaluated, the GA begins evolving the population using the following steps: 1-Selection: Better-performing chromosomes are selected based on their fitness scores using techniques such as tournament or roulette wheel selection.

2-Crossover: Pairs of selected chromosomes are recombined using one-point or uniform crossover to create new offspring, potentially combining beneficial traits from both parents.

3-Mutation: With a small probability, individual genes (recloser locations) are randomly changed to introduce variation and prevent premature convergence. These steps are repeated across several generations, with each iteration moving the population toward better solutions.

### Stopping criteria

**Fig. 1:**
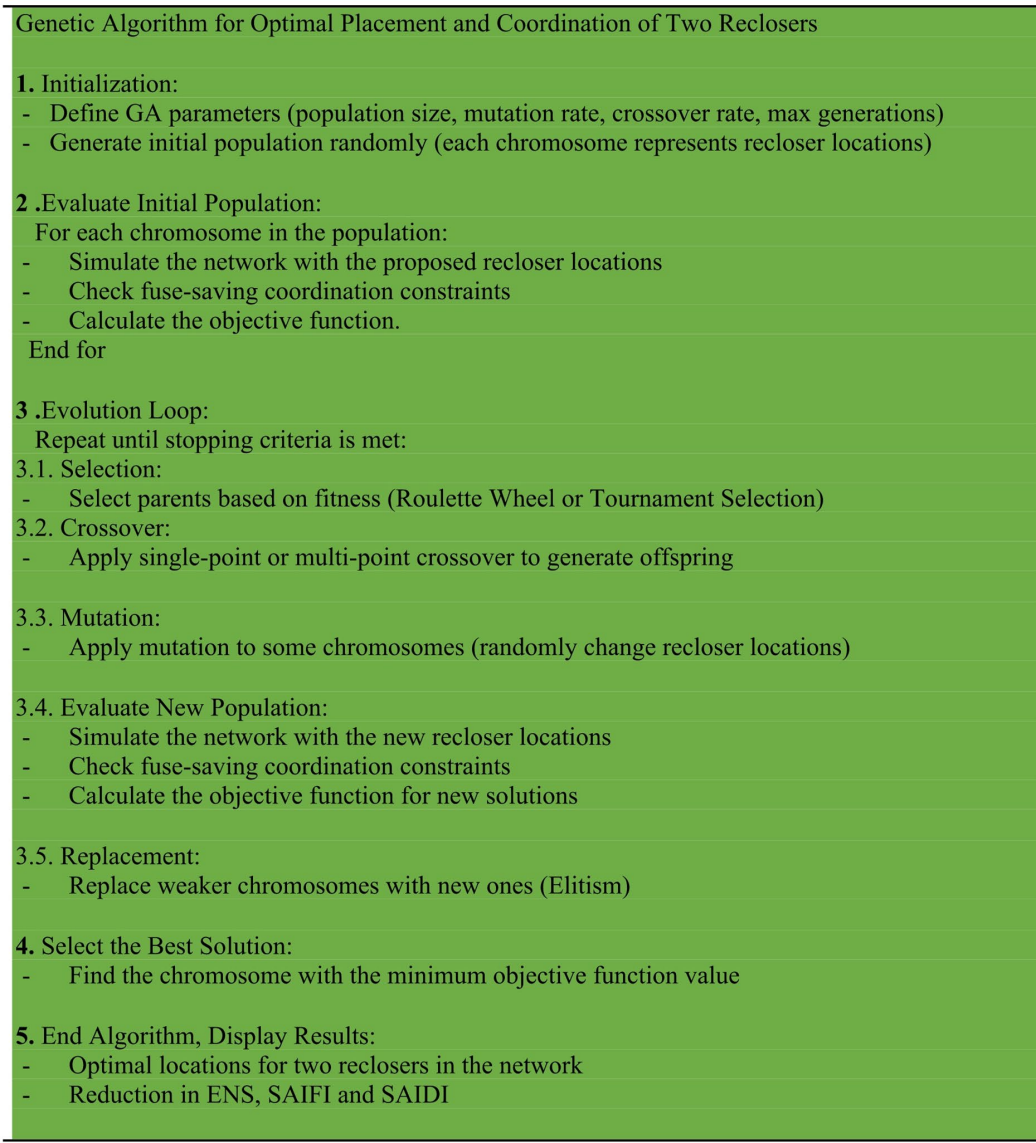
Pseudo-code for the Genetic Optimization Algorithm.

**Fig. 2:**
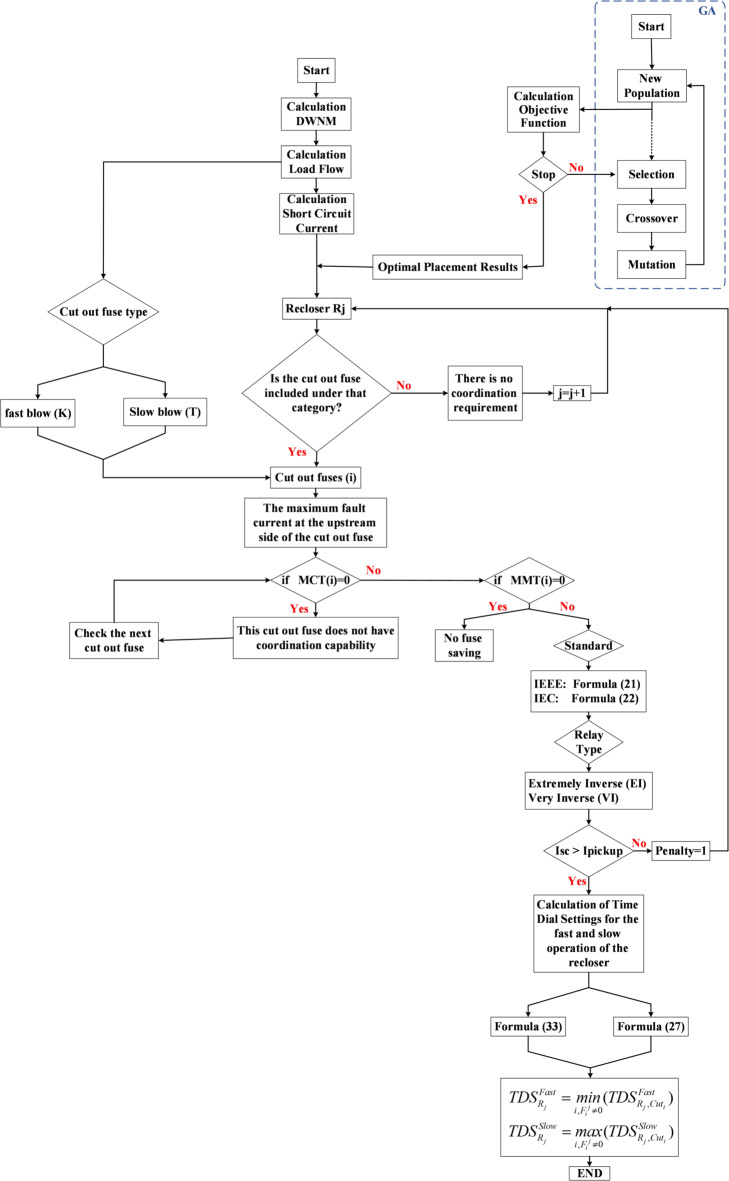
Flowchart of this method.

The algorithm continues until one of the following conditions is met: A fixed number of generations is completed. No significant improvement in the best solution is observed for a certain number of iterations. The fitness value reaches a predefined satisfactory level. Final Output At the end of the process, the chromosome with the best (lowest) objective function value is selected as the optimal solution. This includes: The optimal locations for reclosers, considering fuse-saving coordination. A significant reduction in SAIFI, SAIDI and ENS indicating improved network reliability. A protection scheme that is not only technically feasible but also economically and operationally efficient. This GA-based approach offers a flexible and effective framework for enhancing fault management in modern distribution networks, especially as they evolve toward more intelligent and automated configurations. The pseudo-code for the Genetic Optimization method is provided below Fig. 1:

In the proposed method, a Genetic Algorithm (GA) is first employed to determine the optimal placement of reclosers. In this stage an initial population consisting of a set of possible installation locations is generated, and each solution is evaluated based on reliability indices (ENS, SAIFI, and SAIDI). By applying the selection, crossover and mutation operators new generations are produced and after convergence the optimal locations of the reclosers are obtained. Subsequently protective coordination between the recloser and the fuse is performed, taking the Fuse Saving strategy into account. In this step protective settings including time current characteristic (TCC) curves pickup values, and other operational parameters are determined and evaluated to ensure proper coordination and to prevent unnecessary fuse operations. Finally the proposed method provides the optimal placement of reclosers while satisfying the coordination constraints between the recloser and the cut out fuse. The flowchart of the proposed methodology is presented in Fig. 2.

## Simulation results

In this study, the proposed optimization approach has been evaluated on the standard 33-bus and 69-bus test distribution networks. The simulation results and the performance analysis of the method are presented and discussed in Sect.  4.1 and 4.2 of the manuscript.

### Case study introduction

In this section, the results of applying the proposed methodology to the IEEE 33-bus standard distribution network are presented. The short-circuit capacity at the slack bus is assumed to be 5000 MVA [49], The primary objective of this study is to determine the optimal locations for installing two reclosers in order to minimize reliability indices experienced by customers during switching operations. By optimally allocating these protective devices, the overall reliability and continuity of power supply throughout the distribution network can be significantly improved. In addition to minimizing reliability indices, the coordination between the reclosers and downstream protective fuses is carefully investigated. Proper protection coordination is essential to prevent overlapping protective actions and to ensure selective fault isolation. In the event of a fault, only the affected section of the distribution network should be disconnected, while the remaining healthy parts of the system continue to operate without interruption. In this study, the faults considered in the network are balanced three-phase symmetrical faults with sustained steady-state duration. It is also assumed that the network load remains constant during the entire simulation period to maintain consistent operating conditions. To solve this constrained multi-variable optimization problem, a Genetic Algorithm (GA) is employed due to its robustness and suitability for complex search spaces. The algorithm is evaluated under two different operating scenarios to comprehensively assess its effectiveness. For the GA implementation, an initial population size of 200 and a maximum of 100 generations are selected to achieve an appropriate balance between solution accuracy and computational efficiency. This configuration enables the algorithm to explore a wide range of possible solutions and progressively converge toward the most effective recloser placement strategy.

#### Scenario 1: optimal placement of two reclosers

The optimal placement of two reclosers in the 33-bus network has been carried out. The most suitable locations for the reclosers were identified based on reliability indices, specifically corresponding to lines 6 and 25. Installing the reclosers at these points ensures that, in the event of a fault, the affected section of the network is quickly isolated while the rest of the system remains energized. This rapid response significantly reduces customer outage time, thereby improving the overall reliability of the network. With the reclosers in place, the Energy Not Supplied ENS is reduced to 61.7940 (kWh/yr) the System Average Interruption Frequency Index SAIFI decreases to 8.8750 (int/cus/yr) and the System Average Interruption Duration Index SAIDI drops to 17.75 (h/cus/year). Compared to the base case ENS improves by 48.34%, SAIFI by 44.87% and SAIDI by 44.87% indicating a substantial enhancement in the stability and dependability of the network, as shown in Table 2.

**Table 2:** Reliability Indices with Optimal Placement of Two Reclosers.

Objective	Value	Improvement%
*ENS*_*base*_ *(MWh/yr)*	119.6230	0
*SAIFI*_*base*_ *(int/cus/yr)*	16.10	0
*SAIDI*_*base*_ *(h/cus/yr)*	32.20	0
*ENS (MWh/yr)*	61.7940	48.34%
*SAIFI (int/cus/yr)*	8.8750	44.89%
*SAIDI (h/cus/yr)*	17.78	44.89%

#### Scenario 2: optimal placement of two reclosers with cut out fuses

The optimal placement of two reclosers in the network was done after installing four cutout fuses in lines 18, 22, 10, and 30. The reclosers were optimally placed in lines 6 and 25. This coordination between the fuses and reclosers ensures that in the event of a fault in any line, the faulty section is quickly isolated and the disturbance is prevented from spreading to other parts of the network. While cutout fuses provide fast and localized protection, reclosers enable rapid power recovery after the fault is cleared. This coordination reduces the duration of outages for subscribers, maintains power supply in healthy lines, and ultimately increases the overall reliability of the network. In addition, the optimal placement of reclosers leads to improved reliability indicators and enhances the performance of the network in the face of possible faults.

In the baseline state, the network equipped with cut out fuses had ENS of 73.2590 (kWh/year) SAIFI of 10.0063 (int/cus/yr) and SAIDI of 20.0125 (h/cus/yr) which indicate the level of disruption in the electricity supply to subscribers. Also installing four cut out fuses in the network improved the reliability indices compared to without protective equipment, with the ENS index improving by 38.75% and the SAIFI and SAIDI indices improving by 37.85%. After the optimal installation of two reclosers in lines 6 and 25, the ENS index reached 36.3320 (kWh/year) and SAIFI decreased to 5.3438 (int/cus/yr) and SAIDI reached 10.6875 (h/cus/yr). These improvements are due to the fast and coordinated operation of reclosers and fuses, which isolate the faulty sections and prevent the spread of the disturbance to other sections. Reclosers are able to restore the current after the fault is cleared, while cutout fuses provide fast and localized protection. This coordinated interaction reduces the duration of the outages for customers, maintains the supply of healthy lines, and ultimately improves the reliability of the system.

From a technical point of view, the reduction of reliability indicators and the possibility of fast power recovery indicate a significant increase in the stability and reliability of the distribution network in the face of possible faults, as presented in Table 3.

**Table 3:** Reliability indices with optimal placement of two reclosers in the presence of cut out fuses.

Objective	Value	Improvement%
*ENS*_*base*_ *(MWh/yr)*	73.2590	0
*SAIFI*_*base*_ *(int/cus/yr)*	10.0063	0
*SAIDI*_*base*_ *(h/cus/yr)*	20.0125	0
*ENS (MWh/yr)*	36.3320	50.41%
*SAIFI (int/cus/yr)*	5.3438	46.60%
*SAIDI (h/cus/yr)*	10.6875	46.60%

**Fig. 3:**
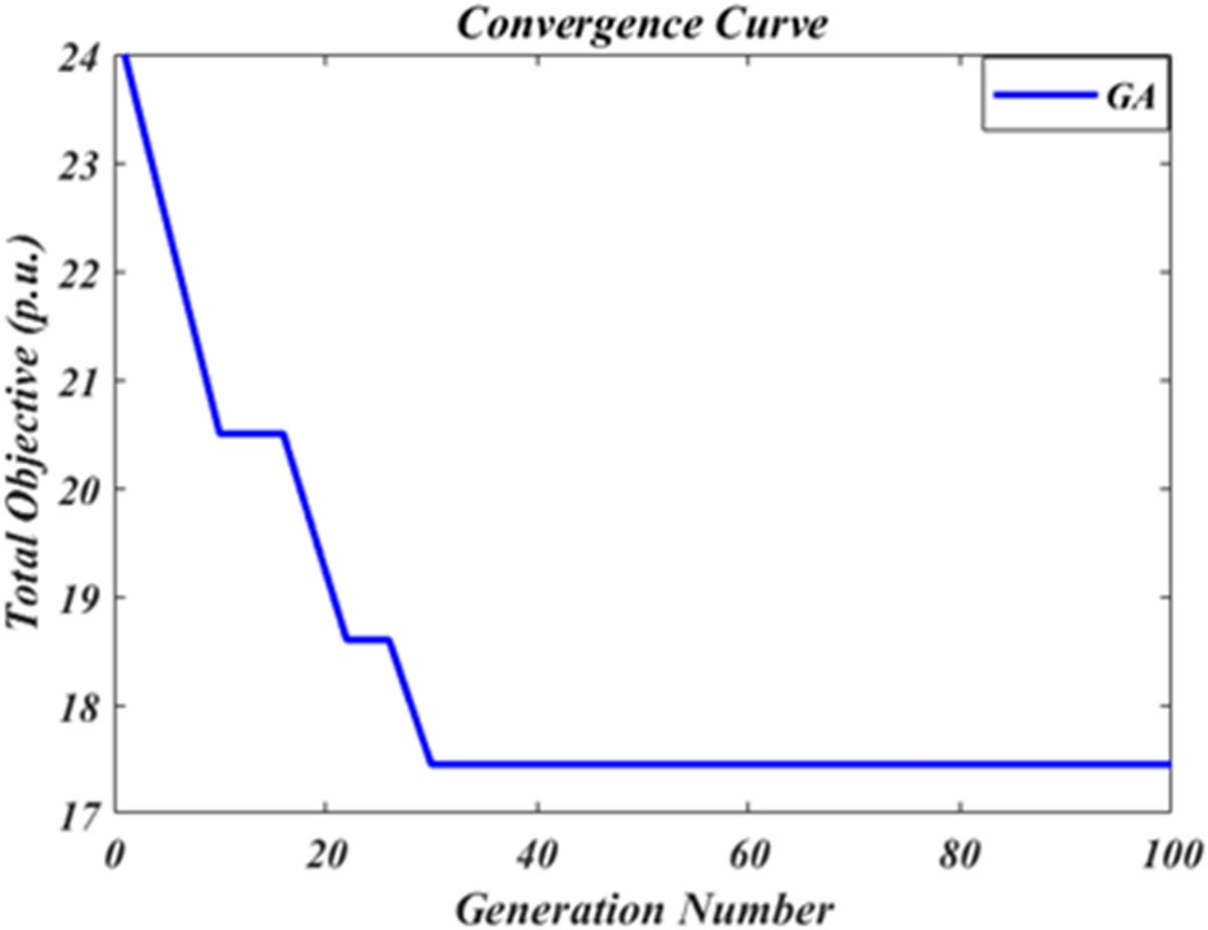
Convergence curve of the proposed method in optimizing the total objective function of reliability indices.

The convergence curve of the reliability indices objective function obtained using the proposed method over 100 independent runs is presented in Fig. 3. As shown in the figure, the proposed method converges to the optimal solution from the 30th generation onward demonstrating its efficiency and stability in achieving consistent results across multiple executions. In this study the weights of the reliability indices (ENS, SAIFI and SAIDI) were considered equal in the objective function, and the overall objective function was also plotted. This behavior not only confirms the algorithms effectiveness in exploring the solution space but also highlights its ability to consistently identify high quality solutions with minimal computational effort. Such convergence characteristics emphasize the robustness and reliability of the proposed method in solving the optimization problem under different initial conditions.

#### Protective coordination of recloser with downstream cutout fuses

To ensure the coordination of the recloser with downstream cutout fuses, the rated current of the cut out fuses must be determined. Based on the load current at the installation locations of the cutout fuses, the rated current values are obtained and presented in Table 4.

**Table 4:** Rated current of installed cutout fuses in the network.

Fuse Number	Line Number	Line load current (A)	Fuse rated current (A)
*F1*	*18*	*18.0871*	*25*
*F2*	*22*	*48.4819*	*65*
*F3*	*10*	*30.6398*	*40*
*F4*	*30*	*23.3488*	*40*

#### Optimal placement of two reclosers in the network

The optimal placement for the two reclosers in the network is on lines 6 and 25. The reclosers set current is determined based on the load current of the line where the recloser is installed. The corresponding values are presented in Table 5.

**Table 5:** Recloser set current values.

Protective Device	Optimal Placement	Load Current (A)	Pickup Current (A)
*R*_*1*_	*6*	*58.3870*	*75.9031*
*R*_*2*_	*25*	*65.3511*	*84.9564*

#### Time coordination settings for reclosers

In this study, the fixed parameters of the relay characteristic according to Eqs. (21) and (22), are considered based on the relay types Very Inverse (VI) and Extremely Inverse (EI) for IEEE and IEC standards, as presented in Table 6 [45]- [46].

**Table 6:** The values of the fixed parameters for VI and EI relay characteristics.

standard	VI			EI		
	A	B	*P*	A	B	*P*
***IEEE***	*19.61*	*0.491*	*2*	*28.2*	*0.1217*	*2*
***IEC***	*13.5*	*1*	*---*	*80*	*2*	*---*

The coordination time intervals of reclosers in both fast and slow modes for type T and K cut out fuses are presented in Table 7.

**Table 7:** Coordination time interval values.

Fuse type	CTI	
	Fast Mode (ms)	Slow Mode (ms)
***T Fuse type***	*0.15*	*0.05*
***K Fuse type***	*0.15*	*0.02*

The time dial settings of the reclosers in both fast and slow modes for based on type T and K cutout fuses and IEEE and IEC standards for Very Inverse (VI) and Extremely Inverse (EI) characteristic curves are shown in Table 8.

**Table 8:** Time Dial Settings For Reclosers Values.

*Recloser*	*Fuse Type*	*Time Dial Setting*	*IEEE*		*IEC*	
			*VI*	*EI*	*VI*	*EI*
***Recloser 1***	***Fuse type T***	$\:{TDS}^{f}$	*0.0606*	*0.1427*	*0.0367*	*0.01*
		$\:{TDS}^{s}$	*0.4638*	*1.0922*	*0.2806*	*0.7654*
	***Fuse type K***	$\:{TDS}^{f}$	*0.0154*	*0.0363*	*0.0093*	*0.0255*
		$\:{TDS}^{s}$	*0.3490*	*0.8219*	*0.2112*	*0.5760*
***Recloser 2***	***Fuse type T***	$\:{TDS}^{f}$	*0.0496*	*0.1103*	*0.0283*	*0.0716*
		$\:{TDS}^{s}$	*0.4372*	*0.9713*	*0.2491*	*0.6306*
	***Fuse type K***	$\:{TDS}^{f}$	*0.0116*	*0.0257*	*0.0069*	*0.0167*
		$\:{TDS}^{s}$	*0.3365*	*0.7474*	*0.1990*	*0.4853*

#### Examination of protective coordination with short circuit at the downstream of cutout fuses F3 and F4

**Fig. 4:**
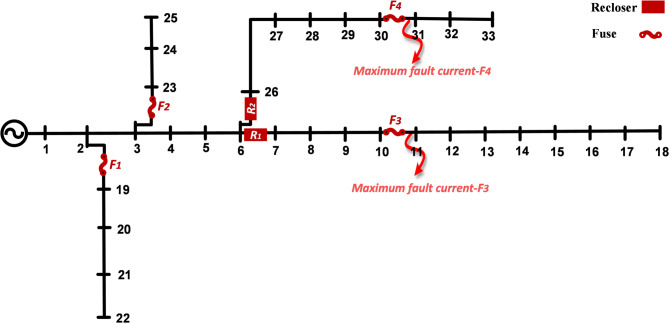
The 33-bus network diagram with protective devices.

In this section, the type of cutout fuses for slow-blow and fast-blow characteristics in the network is analyzed. The IEEE and IEC standards employ the VI characteristic curve. As shown in Fig. 4, with a short circuit occurring upstream of cutout fuse F3, the maximum short-circuit current from the source to the fault location is observed. The short-circuit current path is through recloser R1 and cutout fuse F3. According to Fig. 4, with a short circuit downstream of cutout fuse F4, the short-circuit current path is through recloser R2 and cutout fuse F4.

Network reliability is enhanced through optimal protective coordination between reclosers and cut out fuses. In the event of a short circuit the cutout fuses rapidly isolate the faulted section and prevent the disturbance from spreading to other lines while the reclosers enable fast restoration of power after the fault is cleared. This coordinated operation reduces outage duration for customers maintains supply to healthy lines and improves the network reliability indices. The integrated interaction and rapid response of the protective devices effectively decrease power interruption time and enhance system reliability indices which are fundamental criteria for evaluating the stability and dependability of the distribution network under potential fault conditions.

**Fig. 5:**
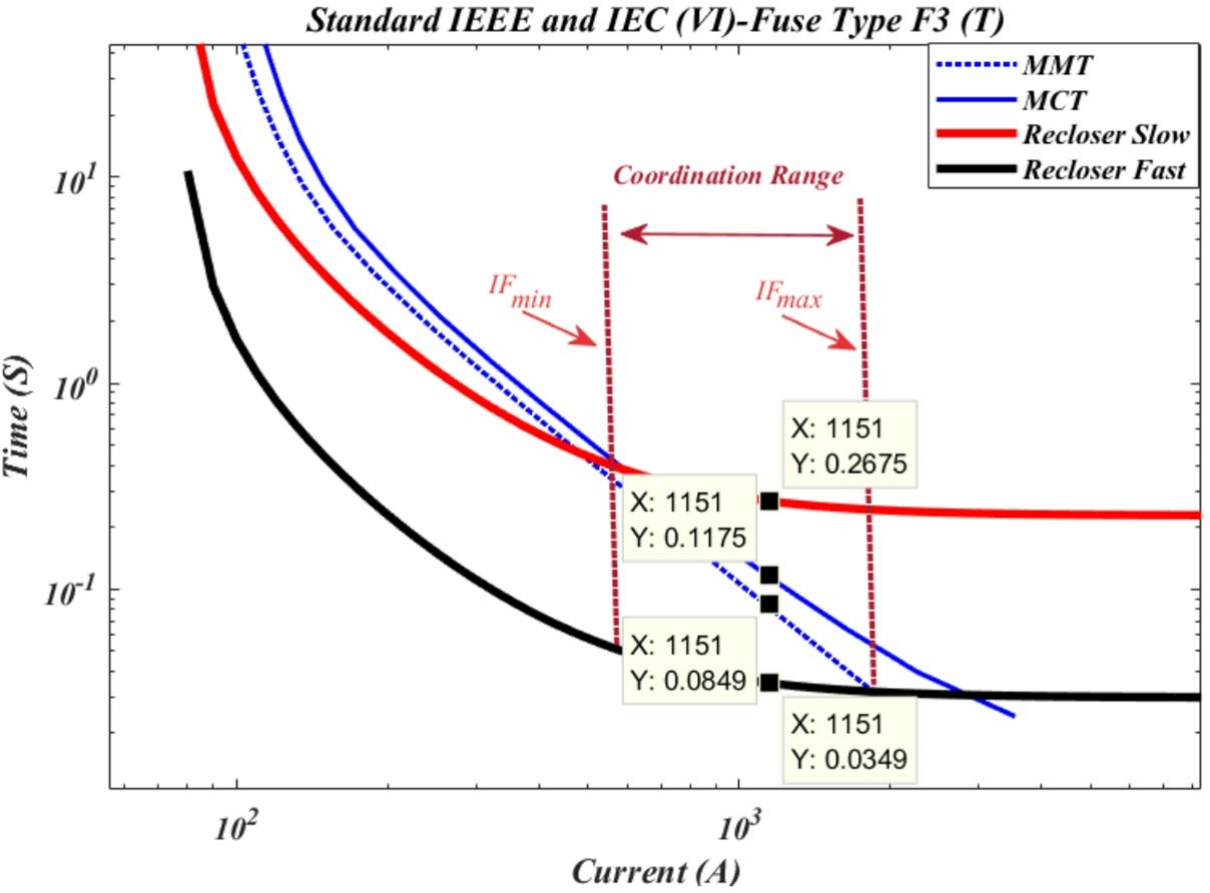
Time current curve of recloser R1 with cut-out fuse F3 of type T.

According to Fig. 5, the maximum short-circuit current downstream of cutout fuse F3 is 1151 A. When a short circuit occurs downstream of cutout fuse F3, the recloser’s operation time in fast mode is 0.0349 s, which is faster than the time-current curve of the cutout fuse. Meanwhile, the operation time of the cutout fuse based on the minimum fuse melting curve is 0.0849 s, and based on the maximum fuse clearing curve, it is 0.1175 s. Additionally, the operation time of the recloser in slow mode is 0.2675 s. The coordination range between the protective devices is from I^F^_min_ to I^F^_max_.

**Fig. 6:**
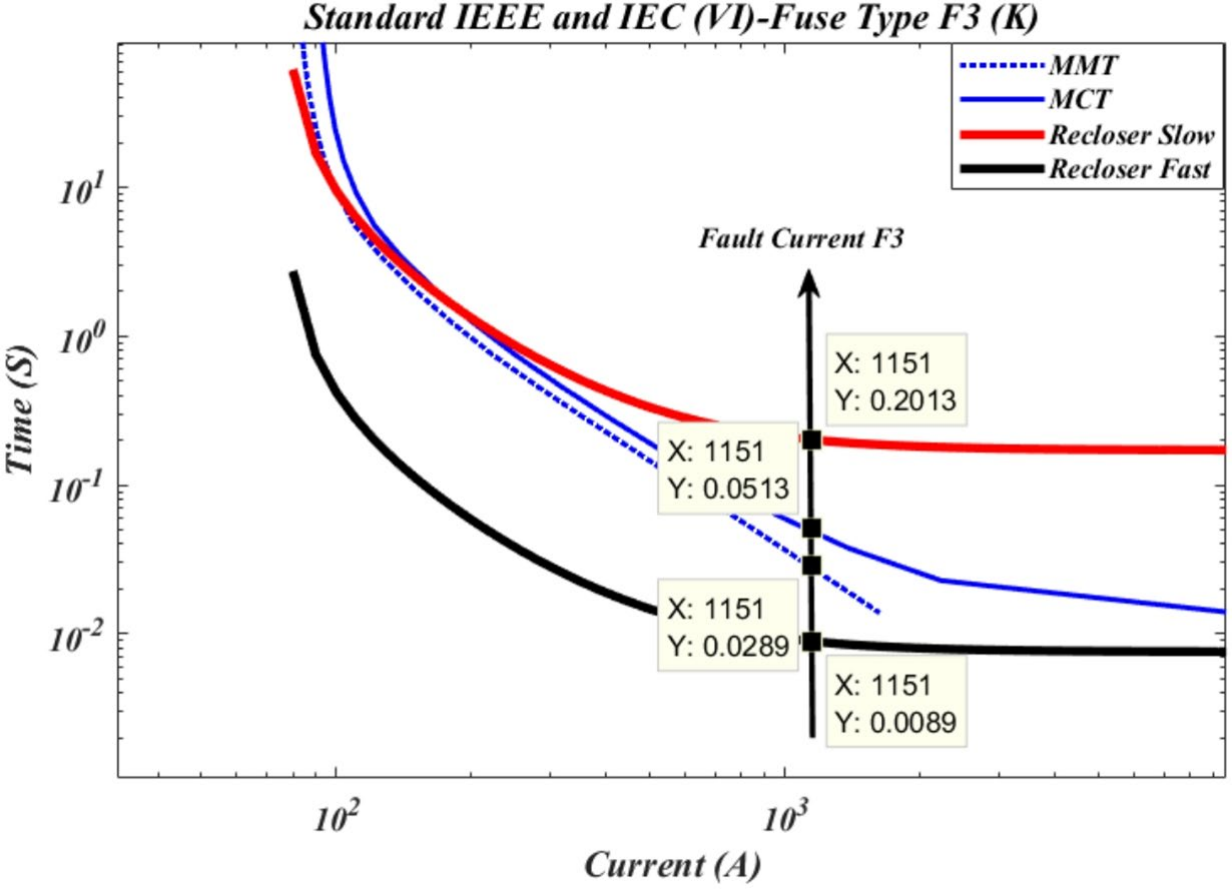
Time current curve of recloser R1 with cut-out fuse F3 of type k.

According to Fig. 6, the maximum short-circuit current downstream of cutout fuse F3 is 1151 A. In the event of a short circuit at this point, the recloser operates in fast mode with an operation time of 0.0089 s, which is faster than the time-current curve of the cutout fuse. The operation time of the cutout fuse based on the minimum fuse melting curve is 0.0289 s, and based on the maximum fuse clearing curve, it is 0.0513 s. Furthermore, the recloser operates in slow mode with an operation time of 0.2013 s.

**Fig. 7:**
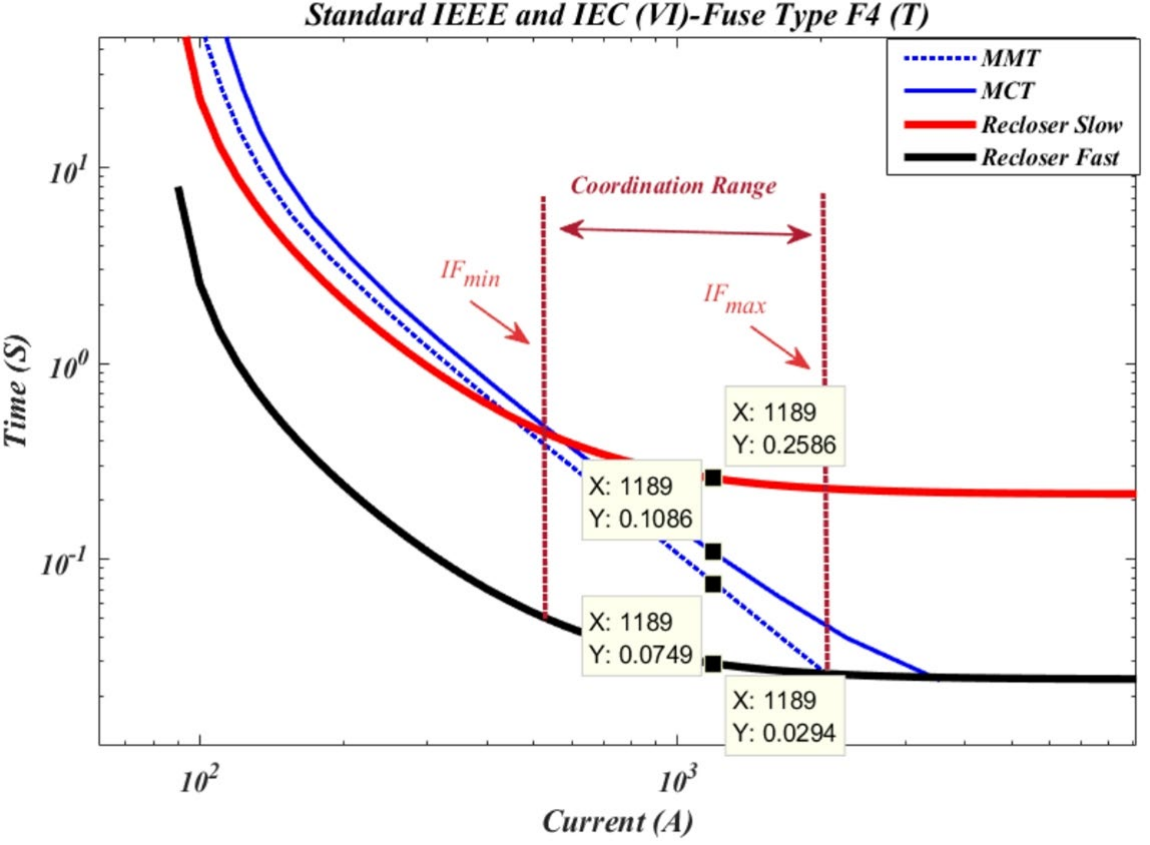
Time current curve of recloser R2 with cut-out fuse F4 of type T.

According to Fig. 7, the maximum short-circuit current downstream of cutout fuse F3 is 1189 A. When a short circuit occurs at this point, the recloser operates in fast mode with a time of 0.0294 s, which is shorter than the time-current curve of the cutout fuse. In contrast, the operation time of the cutout fuse based on the minimum fuse melting curve is 0.0749 s, and based on the maximum fuse clearing curve, it is 0.1086 s. Additionally, the recloser’s operation in slow mode occurs at 0.2586 s. The coordination range between the protective devices is within the range between I^F^_min_ and I^F^_max_.

**Fig. 8:**
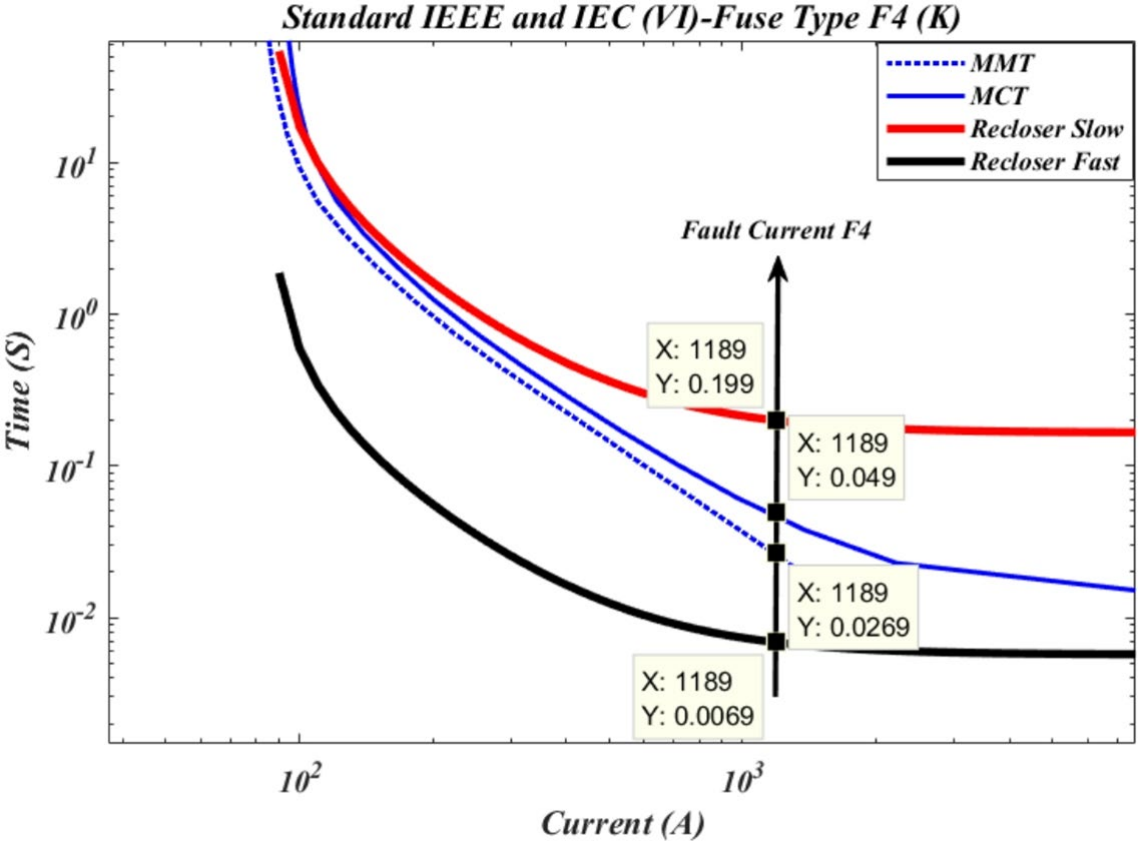
Time current curve of recloser R2 with cut-out fuse F4 of type k.

According to Fig. 8, the maximum short-circuit current downstream of cutout fuse F3 remains 1189 A. In this case, the recloser operates in fast mode with an operation time of 0.0069 s, which is faster than the time-current curve of the cutout fuse. The operation time of the cutout fuse based on the minimum fuse melting curve is 0.0269 s, and based on the maximum fuse clearing curve, it is 0.049 s. Furthermore, in slow mode, the recloser operates with an operation time of 0.199 s.

In order to compare the results and summarize the explanations provided, the results of Scenario 2 are presented in Table 9.

**Table 9:** Coordination Settings Based on IEEE, IEC (VI) Standards for Cut out Fuses F3 and F4.

*Protective Device*	*Short Circuit Current (A)*	*R*_*1*_		*R2*		*Fuse type T*		*Fuse type K*		*Fuse Saving*
		$\:{{O}{T}}^{{f}}$	$\:{{O}{T}}^{{s}}$	$\:{{O}{T}}^{{f}}$	$\:{{O}{T}}^{{s}}$	$\:{{F}{u}{s}{e}}_{{i}}$ ***(*** $\:{{t}}^{{M}{M}{T}}$ ***)***	$\:{{F}{u}{s}{e}}_{{i}}$ ***(*** $\:{{t}}^{{M}{C}{T}}$ ***)***	$\:{{F}{u}{s}{e}}_{{i}}$ ***(*** $\:{{t}}^{{M}{M}{T}}$ ***)***	$\:{{F}{u}{s}{e}}_{{i}}$ ***(*** $\:{{t}}^{{M}{C}{T}}$ ***)***	
***F3***	*1151*	*0.0349*	*0.2675*	*---*	*---*	*0.0849*	*0.1175*	*---*	*---*	*✓*
		*0.0089*	*0.2013*	*---*	*---*	*---*	*---*	*0.0289*	*0.0513*	*✓*
***F4***	*1189*	*---*	*---*	*0.0294*	*0.2586*	*0.0749*	*0.1086*	*---*	*---*	*✓*
		*---*	*---*	*0.0069*	*0.1990*	*---*	*---*	*0.0269*	*0.0490*	*✓*

### 69-bus test network

**Fig. 9:**
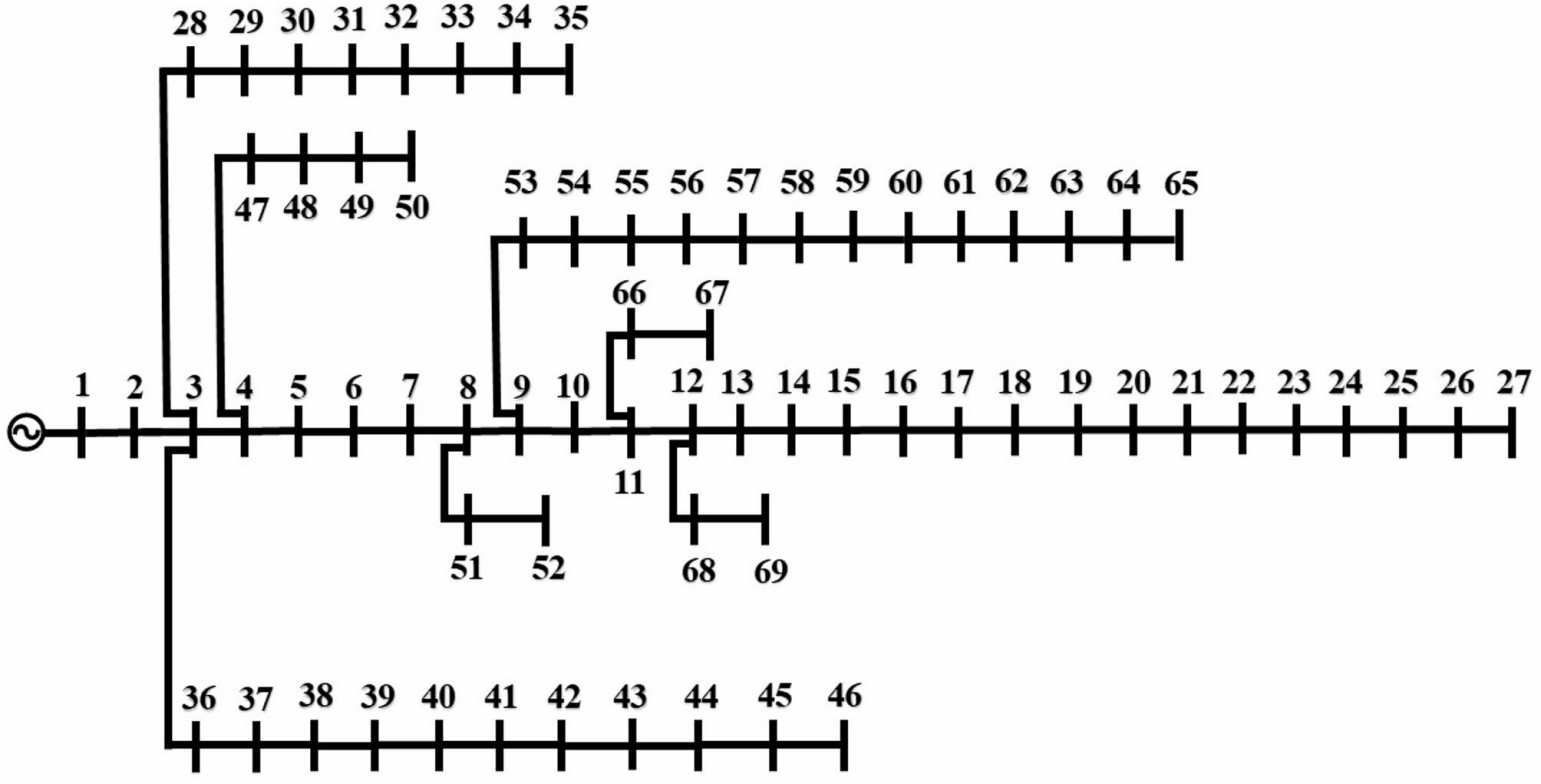
The 69-bus network diagram.

In this section, the results of applying the proposed method to the IEEE 69-bus standard distribution network are presented and illustrated in Fig. 9 [35].

In this section, the study investigates the optimal placement of reclosers in the standard 33-bus and 69-bus distribution networks. The analysis focuses on the combined impact of reclosers and cutout fuses on the reliability indices, which are considered key performance indicators in distribution systems. At each stage, reclosers are incrementally added to the network and the resulting reliability indices are evaluated to determine the extent of improvement in system reliability. The optimal placement strategy is specifically designed to enhance the reliability performance of the network. This approach highlights the importance of optimally locating protective devices in order to improve distribution network performance and strengthen the overall reliability of the system under fault conditions.

#### Optimal placement of recloser

In the 69-bus distribution network, the reliability indices under the base-case condition, without any protective devices, are relatively high. In this state, the Energy Not Supplied (ENS) is 260.8241 (MWh/yr) the System Average Interruption Frequency Index (SAIFI) is 34.30 (int/cus/yr) and the System Average Interruption Duration Index (SAIDI) is 68.60 (h/cus/yr).

To enhance the reliability performance of the network, six cutout fuses were installed on lines 27, 35, 46, 50, 52, and 65. As a result of this installation, ENS went to 133.9788 (MWh/yr), SAIFI went to 16.7188 (int/cus/yr) and SAIDI went to 33.4375 (h/cus/yr). A comparison with the base case clearly demonstrates that integrating cutout fuses has a substantial positive effect on the reliability indices of the network.

In an alternative setup, four cutout fuses were installed on lines 27, 35, 46, and 50. Under this arrangement, the network showed ENS of 165.4464 (MWh/yr), SAIFI of 22.5688 (int/cus/yr) and SAIDI of 45.1375 (h/cus/yr). While keeping these four fuses in place, an optimization procedure was performed to identify the best location for a single recloser. The results indicated that line 9 is the most suitable position. After placing the recloser at this line, ENS went to 104.1536 (MWh/yr), SAIFI went to 16.2562 (int/cus/yr) and SAIDI went to 32.5125 (h/cus/yr). These outcomes clearly demonstrate that proper recloser placement in distribution systems plays a crucial role in strengthening reliability. By isolating temporary faults and restoring service automatically when possible, the recloser helps control both the frequency and duration of interruptions, thereby enhancing overall network performance.

The comprehensive results of optimal recloser placement for the 33-bus and 69-bus networks are summarized in Table 10. In this study, reclosers were deployed step by step using an optimization algorithm to achieve the maximum possible improvement in reliability indices. The findings show that each additional recloser, when installed at its most effective location, contributes significantly to outage mitigation and system stability. Overall, the coordinated and optimal deployment of reclosers and cutout fuses leads to a substantial enhancement in network reliability, highlighting the importance of strategic protective device placement in distribution systems.

**Table 10:** Optimal placement of recloser in standard 33-bus and 69-bus networks.

*Test Network*	*Recloser number*	*Cut out fuse number*	*Line number of* *cut out fuse*	*Optimal placement*	*Reliability Indices*		
					*ENS (MWh/yr)*	*SAIFI (int/cus/yr)*	*SAIDI (h/cus/yr)*
***33-bus***	*---*	*---*	*---*	*---*	*119.6230*	*16.10*	*32.20*
	*---*	*4*	*18*,*22*,*10*,*30*	*---*	*73.2590*	*10.0063*	*17.75*
	*1*	*4*	*18*,*22*,*10*,*30*	*Line 4*	*49.2590*	*7.5313*	*15.0625*
	*2*	*4*	*18*,*22*,*10*,*30*	*Lines 6*,* 25*	*36.3320*	*5.3435*	*10.6875*
	*3*	*4*	*18*,*22*,*10*,*30*	*Lines 3*,* 6*,* 25*	*33.9640*	*5.1188*	*10.2375*
***69-bus***	*---*	*---*	*---*	*---*	*260.8241*	*34.30*	*68.60*
	*---*	*6*	*27*,*35*,*46*,*50*,*52*,*65*	*---*	*133.9788*	*16.7188*	*33.4375*
	*---*	*8*	*20*,*27*,*35*,*46*,*50*,*52*,*65*,*67*	*---*	*103.9288*	*12.9646*	*25.9292*
	*1*	*4*	*27*,*35*,*46*,*50*	*Line 9*	*104.1536*	*16.2562*	*32.5125*
	*2*	*6*	*27*,*35*,*46*,*50*,*52*,*65*	*Lines 4*,* 9*	*69.6623*	*9.3771*	*18.7542*
	*3*	*6*	*27*,*35*,*46*,*50*,*52*,*65*	*Lines 4*,* 9*,* 12*	*64.0863*	*8.5937*	*17.1875*

## Conclusion

The results of this study demonstrate that the optimal placement of reclosers, combined with coordinated operation alongside cutout fuses, plays a pivotal role in enhancing the reliability of distribution networks. Implementing a fuse saving strategy and precisely configuring the time current characteristic (TCC) curves enables reclosers to effectively isolate faulted sections while preventing unnecessary fuse operations, thereby reducing both the frequency and duration of outages and improving service continuity. In the 33-bus network with four cutout fuses installed and two reclosers optimally placed on lines 6 and 25, the reliability indices improved significantly, the Energy Not Supplied (ENS) was 36.3320 (MWh/yr) the System Average Interruption Frequency Index (SAIFI) was 5.3435 (int/cus/yr) and the System Average Interruption Duration Index (SAIDI) was 106.875 (h/cus/yr). Optimal placement of the reclosers allows faulted sections to be quickly isolated while avoiding unnecessary fuse operations. This coordination not only reduces both temporary and permanent outages but also ensures that protective actions are executed selectively and in harmony, thereby enhancing overall network reliability. Additionally fine tuning the operating characteristics of protective devices optimizes network response times and prevents superfluous operations, further improving the overall performance of the protection system. For the 69-bus network with four cutout fuses installed and one recloser optimally located on line 9, reliability indices also improved considerably ENS decreased to 104.1536 (MWh/yr), SAIFI to 16.2562 (int/cus/yr) and SAIDI to 32.5125 (h/cus/yr). Optimal recloser placement ensures rapid isolation of faulted sections, prevents unnecessary fuse operations, and guarantees coordinated and selective protective actions, resulting in reduced outages and enhanced reliability. Overall the findings highlight that a combined strategy of optimal placement and setting of protective devices together with precise coordination and the avoidance of unnecessary fuse operations, represents an effective approach for enhancing both the reliability and operational efficiency of distribution systems.

### Future work

#### Recloser–recloser coordination

Future research should focus on the detailed coordination of multiple series connected reclosers in distribution networks. Proper adjustment of the Time–Current Characteristic (TCC) curves is essential to ensure selectivity such that the downstream recloser operates first, and the upstream recloser acts only if the downstream device fails to clear the fault. Well designed TCC coordination prevents the simultaneous operation of protective devices, minimizes unnecessary outages, and significantly enhances the overall reliability and operational security of the distribution system.

#### Validation of coordination margins under dynamic conditions

Another important direction for future research is the validation of coordination margins of protective devices under dynamic and real operating conditions. While many existing studies assume static network parameters, real-world distribution systems experience continuous variations in load demand, network topology and the intermittent behavior of distributed generation. These variations can significantly influence short circuit levels operating times and coordination margins of protective devices. Therefore dynamic validation of protection settings is essential to ensure robust and reliable performance under practical operating scenarios.

## Data Availability

Data will be available on reasonable request (email: Me.hajiabadi@hsu.ac.ir).

## Abbreviations

L: Number of branches
N: Number of nodes
S: Supplying point
$T =[t_{n,l}]$: node and line incidence matrix
$$M = [m_{i,j}]$$: Adjacency matrix
DWNM: Down Warding Node Matrix
BIBC: bus injection to branch current
BCBV: branch current to bus voltage
$[I_b]_{l,1}$: the current passing through each line
$[V_n]_{n+1}$: the voltage of the nodes
$[I_k^f]_{N \times 1}$: the short circuit current at each bus k
Z_kk_: impedance matrix
Z_f_: fault impedance
[V_n_^f^]: The bus voltages during the fault
VI: Very Inverse
ENS: Energy Not Supplied
EI: Extremely Inverse
I_sc_: Line short circuit current during fault
I_pickp_: pickup curretn
MMT: Minimum Melting Time
MCT: Maximum Clearing Time
OT^ieee^: Relay operating time based on IEEE standard
OT^iec^: Relay operating time based on iec standard
TDS: time dial setting
CTI: Coordination Time Interval
FS: fuse saving
L_n_: load at load point n
U_n_: Average outage duration at load point n
$\lambda_l$: failure rate of the line
t_l,n_: repair time of the line
X_l,n_: Lines whose failure causes an outage at load point n
